# One-year outcomes of bioresorbable magnesium scaffold implantation in complex coronary lesions

**DOI:** 10.3389/fcvm.2026.1854686

**Published:** 2026-06-12

**Authors:** Simon Wölbert, Thomas Schmidt, Fabian Wittek, Benjamin Mayer, Philip Raake, Dario Bongiovanni, Jan Torzewski

**Affiliations:** 1 Department of Cardiology, Cardiovascular Center Oberallgäu-Kempten, Kempten, Germany; 2 Department of Internal Medicine I, University Hospital Augsburg, Augsburg, Germany; 3 Institute of Epidemiology and Medical Biometry, University of Ulm, Ulm, Germany

**Keywords:** bioresorbable scaffold, coronary artery disease, complex coronary lesions, *de novo* lesions, ischemic heart disease, Magmaris

## Abstract

**Background:**

Magmaris is a bioresorbable magnesium scaffold that has shown favorable safety and efficacy in the treatment of de-novo coronary lesions.

**Aims:**

To report 1-year outcomes after Magmaris implantation in patients with predominantly complex coronary lesions in the Resorbable Magnesium Scaffold registry (RMS) and to compare them with outcomes from patients with mainly non-complex lesions in the BIOSOLVE-IV registry.

**Methods:**

The RMS registry is an ongoing, prospective, national, multicenter registry conducted in Germany. Enrollment took place from November 2020 until May 2023 at Cardiovascular Center Oberallgaeu-Kempten, Bavaria, Germany. Follow-up was performed by phone 12 months after index procedure. We report 1-year outcomes of the first 100 consecutively enrolled patients and provide an unadjusted statistical comparison with 1-year outcomes from the BIOSOLVE-IV registry.

**Results:**

RMS patients were older (64.9 ± 8.5 vs. 61.9 ± 10.5 years; *p* = 0.006) and had a significantly more complex lesion profile (*p* < 0.001), with higher rates of type B2/C lesions (74.0% vs. 15.1%) and moderate-to-severe calcification (24.0% vs. 7.5%), whereas lesion length was similar between the registries (*p* = 0.118). At 12 months, target-lesion failure did not differ significantly between RMS and BIOSOLVE-IV (*p* = 0.885), while scaffold thrombosis was numerically more frequent in RMS (2.0% vs. 0.8%; *p* = 0.384).

**Conclusion:**

Despite a more complex lesion profile in RMS, no statistically significant differences in 1-year clinical outcomes were observed between the two registries. This analysis is hypothesis-generating though not conclusive. Conclusive analysis can only be provided by a randomized controlled trial.

**Clinical Trial Registration:**

RMS registry, NCT04679740.

## 1 Introduction

Coronary artery disease remains a leading cause of morbidity and mortality worldwide (1). Contemporary drug-eluting stents (DES) represent the state-of-the-art treatment for obstructive coronary artery disease in percutaneous coronary intervention (PCI). Although DES have substantially reduced rates of target lesion failure (TLF) and stent thrombosis (ST) compared with early-generation DES and bare-metal stents (BMS), the permanent metallic stent may still predispose to long-term complications such as late and very late ST (2, 3). The concept of “leaving nothing behind” aims to overcome these limitations, and bioresorbable scaffolds (BRS) are part of this approach. The most extensively studied BRS, the polymer-based ABSORB scaffold, failed to achieve broad clinical adoption due to elevated rates of scaffold thrombosis during the bioresorption phase (4, 5) leading to a Class III (Level of Evidence C) recommendation in the 2018, still valid, ESC/EACTS guidelines on myocardial revascularization (6). The second generation magnesium scaffold device, DREAMS 2G (Drug-Eluting Absorbable Magnesium Scaffold, 2nd Generation; Magmaris), has demonstrated safety and efficacy in the treatment of *de novo* coronary lesions in several registry-based studies (7, 8). However, these studies primarily enrolled non-complex patients with relatively simple coronary lesions. An Italian investigator-initiated registry included a substantial proportion of patients with acute coronary syndromes and more complex coronary lesions. Two-year outcomes demonstrated good safety regarding target lesion failure and, notably, scaffold thrombosis, suggesting that magnesium-based bioresorbable scaffolds may remain of interest in higher-risk subgroups, while further evaluation of newer device generations is warranted (9). In Germany, bioresorbable magnesium scaffold implantation is not fully reimbursed by the health care system, which poses challenges for further technological development. We herein report the 1-year outcomes of the first 100 consecutively enrolled patients from the RMS registry, comprising mostly complex coronary lesions treated with Magmaris, to provide real-world evidence for this population. Being aware of the limitations of such an analysis, we performed an unadjusted statistical comparison with the large-scale BIOSOLVE-IV registry to assess potential outcome differences related to lesion complexity. Preliminary findings from this analysis were presented at the TCT 2025 conference and published as an abstract in the *JACC: Cardiovascular Interventions* Supplementary Appendix (10).

## 2 Methods

### 2.1 Study design and patients

The RMS registry is a prospective, single-arm, multicenter, all-comers registry designed to reflect daily clinical practice and adherence to current guidelines. It was initiated by BIOTRONIK AG. The study cohort of the present analysis comprises the first 100 patients solely enrolled at one center (Cardiovascular Center Oberallgaeu-Kempten, Bavaria, Germany). Patients included in this analysis were consecutively recruited between November 2020 and May 2023. Overall recruitment is still ongoing but has recently transitioned to Freesolve. Although the present registry is part of a larger multicenter program, we deliberately analyzed our single-center experience for three main reasons: first, a single-center analysis ensures consistency in implantation technique, follow-up procedures, and endpoint definitions, thereby reducing heterogeneity. Second, presenting these data provides an early, hypothesis-generating contribution that complements the evidence from multicenter registries. Third, Magmaris has meanwhile been replaced by Freesolve within the RMS registry and is no longer available. Thus, this focused analysis may emphasize clinically relevant aspects that could otherwise be diluted in a broader, more heterogeneous dataset.

Individual patient-level data and 1-year outcomes of BIOSOLVE-IV were obtained directly from BIOTRONIK AG and/or extracted from Wlodarczak et al. (7), where the study design and population are described in detail. A data-sharing agreement was set up for safe data transfer and processing between BIOTRONIK AG and the Cardiovascular Center Oberallgaeu-Kempten.

The datasets generated and/or analyzed during the current study are not publicly available due to patient privacy regulations and restrictions imposed by the data provider (BIOTRONIK AG) but are available from the corresponding author upon reasonable request and with permission of BIOTRONIK AG. S.W. and J.T. had full access to all the data in the study and take responsibility for the integrity of the data and the accuracy of the data analysis.

Beyond providing access to the BIOSOLVE-IV registry data, the sponsor had no role in patient care, event adjudication, data analysis, or manuscript preparation. Cohorts of both studies were strictly separated. Inclusion criteria were similar unless otherwise indicated: (1) proven coronary artery disease; (2) a maximum of two *de novo* lesions in two major epicardial vessels (not mandatory in RMS); (3) target lesion length < 21 mm by visual estimation or quantitative coronary analysis (QCA); target lesion length < scaffold length; (4) reference vessel diameter between 2.7 and 3.7 mm by visual estimation, determining scaffold size. Exclusion criteria were: (1) contraindication to dual antiplatelet therapy (DAPT); (2) left ventricular ejection fraction (LVEF) <30% (not mandatory in RMS); (3) left main lesions (not mandatory in RMS); (4) ostial lesion within 5 mm of the vessel origin; (5) side branch diameter > 2.0 mm originating within the target lesion; (6) STEMI ≤ 72 h before the planned scaffold implantation. A full list of inclusion and exclusion criteria can be found on clinicaltrials.gov (NCT04679740—RMS registry; NCT02817802—BIOSOLVE-IV registry). The study received approval by the Ethics Committee of the Bavarian Medical Association on October 21, 2020 (reference number mb20051). Written informed consent was obtained in accordance with the Declaration of Helsinki, the International Council for Harmonization of Technical Requirements for Pharmaceuticals for Human Use—Good Clinical Practice (ICH-GCP), and the International Organization for Standardization (ISO 14155).

### 2.2 Device and procedure

Magmaris is a bioresorbable magnesium scaffold designed for the treatment of *de novo* coronary artery lesions. It consists of a proprietary magnesium alloy coated with the antiproliferative agent sirolimus. The bioresorption process is completed after approximately 12 months. The implantation strategy follows the 4P principle, proposed by an expert consensus prior to CE mark approval in 2016, which serves as the procedural guideline for this device. It emphasizes adequate pre-dilation (<20% residual stenosis), appropriate patient selection, accurate vessel sizing, and mandatory post-dilation (11). As the RMS registry reflects real-world practice, the use of intracoronary imaging, i.e., intravascular ultrasound (IVUS) or optical coherence tomography (OCT), was left to the operator's discretion. During the index procedure, IVUS/OCT was used in 2 of 104 lesions (1.9%), corresponding to 2 of 100 patients (2%).

### 2.3 Assessment of coronary lesions

Baseline lesion assessment and classification were performed by the study investigators according to the American College of Cardiology/American Heart Association (ACC/AHA) lesion classification system. Complex lesions were defined as those meeting either one Type C or two Type B criteria, consistent with the approach used in BIOSOLVE-IV. Determination was made prospectively at the time of the procedure by the treating interventional cardiologists and retrospectively reviewed by Wölbert S., with validation by the corresponding author (12, 13). Retrospective measurements were conducted by contouring the diseased coronary segments containing the target lesion based on clearly distinguishable gray scale contrast on coronary angiograms. The stepwise angiographic lesion assessment process is illustrated in Supplementary Table S1, demonstrating the standardized approach used for retrospective lesion classification and measurements.

The FUJIFILM Synapse PACS Viewer (version 5.7.242) was used for all measurements. This software enables standardized quantification of lesion length, degree of stenosis, and vessel morphology from digital angiographic images in accordance with the Digital Imaging and Communications in Medicine (DICOM) standard.

### 2.4 Clinical endpoints

Clinical events were adjudicated by the first author, a physician investigator specifically trained in event abstraction and verification according to ARC definitions, under close supervision of experienced interventional cardiologists and validated by the corresponding author.

The primary composite endpoint of the RMS registry was target-lesion failure (TLF) within 1 year of the index procedure. TLF was defined as the composite of cardiac death, target-vessel myocardial infarction (Q-wave or non-Q-wave), and clinically driven target-lesion revascularization. Secondary endpoints comprised the individual components of TLF. Scaffold thrombosis was subclassified as acute (0–24 h after the index procedure), subacute (1–30 days), and late (31 days to 1 year) scaffold thrombosis. All endpoints and scaffold thrombosis definitions followed the most recent Academic Research Consortium (ARC-2) criteria (14). In contrast, BIOSOLVE-IV was initiated before the publication of ARC-2 and therefore relied on previous definitions. Specifically, Wlodarczak et al. (7) used the following: procedural and periprocedural myocardial infarction according to the Society for Cardiovascular Angiography and Interventions (SCAI) (15), spontaneous myocardial infarction according to the Extended Historical Definition (EHD) (16), and clinically-driven target-vessel myocardial infarction and scaffold thrombosis according to ARC-1 (17).

For the present analysis, BIOTRONIK provided TLF event data from BIOSOLVE-IV based on the Third Universal Definition of Myocardial Infarction, which aligns with current ARC recommendations. Although this reclassification slightly increased the absolute number of TLF events in BIOSOLVE-IV compared with the original publication, methodological consistency was ensured. Upon re-evaluation by Wölbert S., including detailed review of procedural and post-procedural clinical visits as well as electrocardiograms (ECG), TLF event rates in RMS remained essentially unchanged even if BIOSOLVE-IV definitions were retrospectively applied. All adjudicated events were subsequently validated by experienced interventional cardiologists to ensure accuracy and consistency. Thus, reasonable comparability of primary endpoint data between both registries was achieved despite differing historical frameworks.

At 1-year follow-up, angina status was assessed by structured telephone interview. Patients were directly asked whether they experienced angina pectoris symptoms, without application of a formal questionnaire. Telephone interviews were conducted by certified study nurses also involved in ABSORB-IV (5), BIOSOLVE-IV (7), BIOMAG-I (18), and BIOMAG-II and were validated by the corresponding author.

### 2.5 Statistical analysis

All data were collected using Microsoft Excel (Microsoft® Excel for Mac, version 16.78). Statistical analyses were performed with IBM SPSS® Statistics, version 29.0.1.0 (IBM Corp., Armonk, NY, USA). Categorical variables are presented as absolute and relative frequencies, whereas continuous variables are expressed as mean ± standard deviation. Comparisons between the two registries were conducted to identify statistically significant differences. The Chi-square test, Fisher's exact test, or Fisher–Freeman–Halton test were applied to compare categorical variables, as appropriate. Trends in left ventricular ejection fraction (LVEF) were assessed using the Cochran–Armitage test for trend, implemented in RStudio (version 2024.12.0 + 467) since this function is not available in SPSS (see Supplementary Material for R code). The R script used for the Cochran–Armitage trend test is provided in Supplementary Appendix*.* For the primary composite endpoint, Kaplan–Meier estimates with 95% confidence intervals were calculated. Survival curves were compared between groups using the log-rank test to detect potential differences in time-to-event rates. The statistical approach to cross-registry comparisons is critically discussed in the section “Limitations of cross-registry comparison from a statistical point of view.” A two-sided *p*-value <0.05 was considered statistically significant for all tests. Figures were created using Microsoft Word (version 16.77.1), Microsoft Excel (version 16.77.1), Lucidchart, or GraphPad Prism (version 10.3.0).

## 3 Results

**Figure 1:**
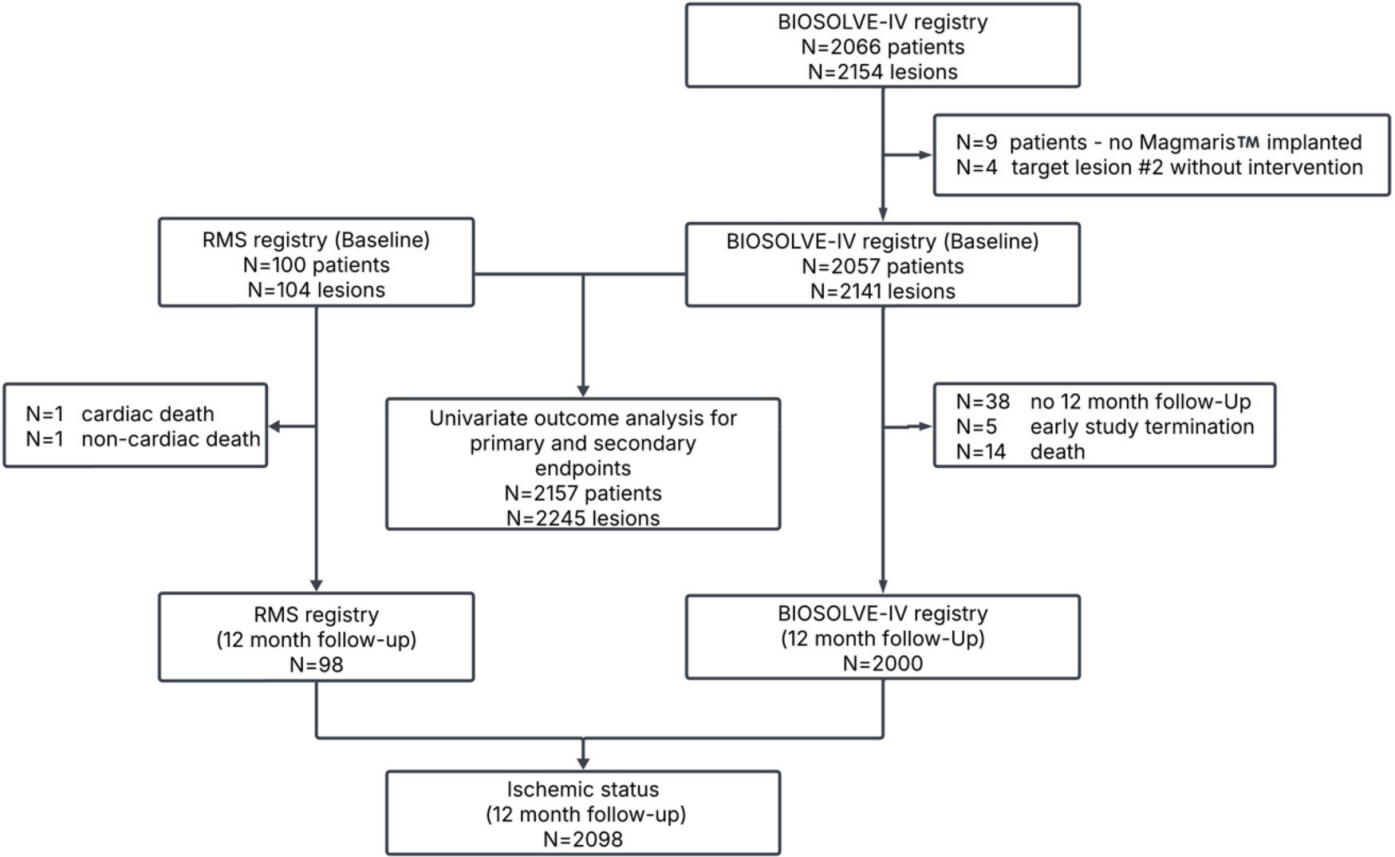
Flow of patient inclusion, exclusions, and availability for 12-month outcome analysis in the RMS and BIOSOLVE-IV registries. Flow of patients and lesions included in the final analysis of the RMS and BIOSOLVE-IV registries. The diagram summarizes patient and lesion numbers at baseline, after exclusions, and after 12-month follow-up. RMS, resorbable magnesium scaffold registry; BIOSOLVE-IV = BIOTRONIK post-marketing registry of the Magmaris scaffold.

Data on composition of both cohorts, follow-up availability, and patients eligible for statistical analysis are displayed in Figure 1.

**Figure 2:**
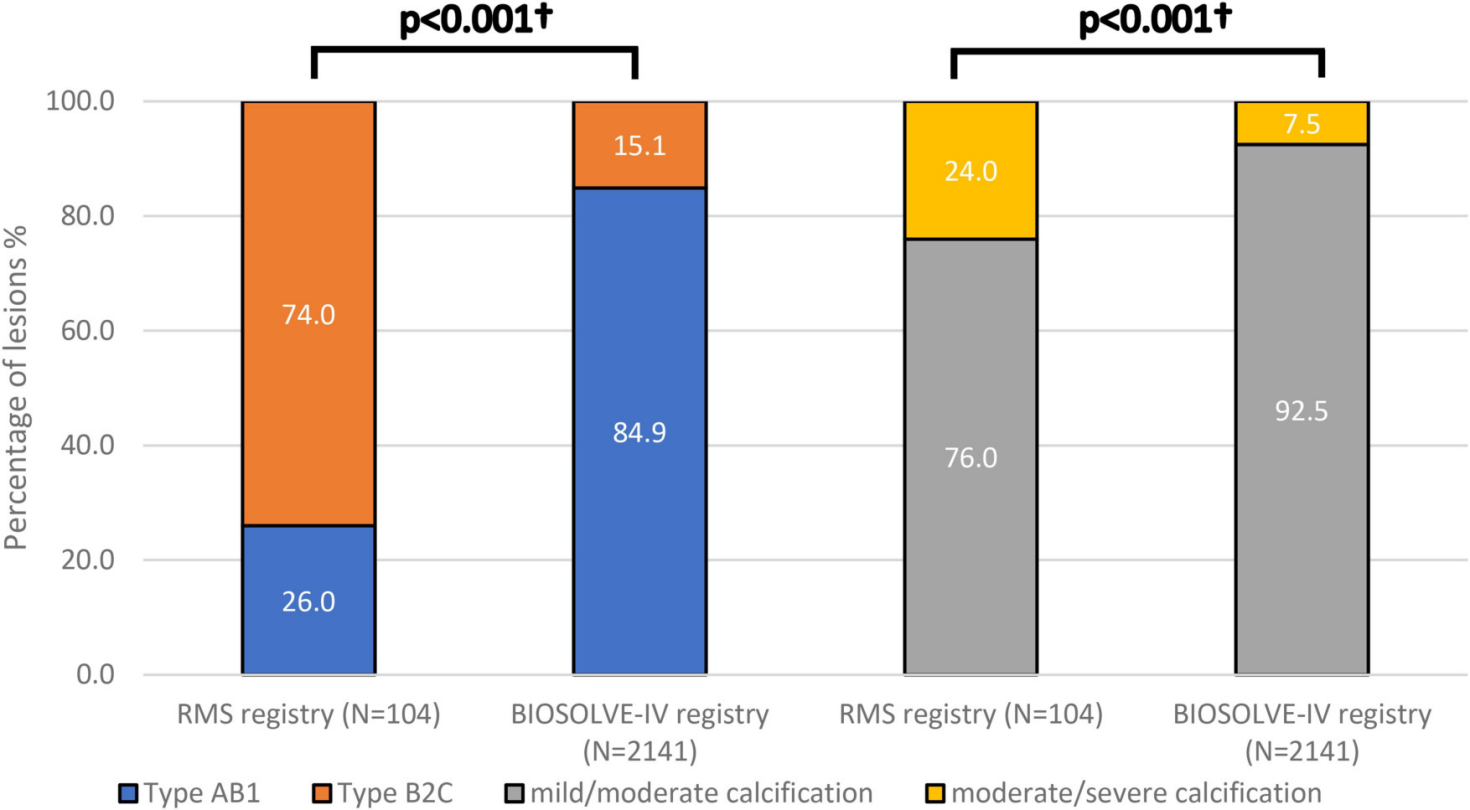
Type B2/C lesions and moderate/severe calcification in RMS vs. BIOSOLVE-IV. Distribution of lesion types (A/B1 vs. B2/C) and prevalence of moderate–severe calcification in the RMS and BIOSOLVE-IV registries. The RMS cohort shows a higher proportion of complex and calcified lesions. ^†^Chi-square test.

Patients were 64.9 ± 8.5 years old in RMS and 61.9 ± 10.5 years old in BIOSOLVE-IV (*p* = 0.006). Twenty-six percent of patients in RMS and 21.6% of patients in BIOSOLVE-IV had diabetes mellitus (*p* = 0.297). In the RMS cohort, insulin-dependent diabetes was more common than in BIOSOLVE-IV. While lesion length and target vessel distribution were largely comparable between the registries, lesions treated in RMS were angiographically more severe and were more often complex and moderately to severely calcified (Table 1 for baseline characteristics, Table 2 for lesion characteristics, Figure 2 for lesion calcification). Adequate lesion preparation and post-dilation in accordance with guideline recommendations were performed in nearly all interventions in both studies (Table 2). In the RMS cohort, intracoronary imaging was performed in 2%, whereas corresponding data were not available for BIOSOLVE-IV.

**Table 1:** Baseline characteristics RMS and BIOSOLVE-IV.

Patient characteristics	RMS	BIOSOLVE-IV	*p*-value
	*N* = 100	*N* = 2,057	
Epidemiological data			
Age	64.9 ± 8.5	61.9 ± 10.5	0.006^c^
Male	78 (78)	1,531 (74.4)	0.423^a^
Cardiovascular risk factors			
Arterial hypertension	73 (73)	1,365 (66.4)	0.169^a^
Dyslipidaemia	49 (49)	1,340 (65.1)	<0.001^a^
Diabetes mellitus	26 (26)	444 (21.6)	0.296^a^
Insulin-dependent^d^	10 (38.5)	94 (21.1)	0.039^a^
Smoking history	58 (58)	1,224 (59.2)	0.779^a^
Active smoking^d^	26 (44.8)	509 (41.9)	0.647^a^
Chronic kidney disease	4 (4)	125 (6.1)	0.392^a^
Ischemic status			
			<0.001^a^
Stable angina pectoris	28 (28)	994 (48.3)	
Unstable angina pectoris	16 (16)	358 (17.4)	
Silent ischemia	29 (29)	316 (15.4)	
NSTE-ACS	26 (26)	381 (18.5)	
STEMI	1 (1)	8 (0.4)	
Cardiac function and prior cardiac history			
Prior PCI	35 (35)	589 (28.6)	0.170^a^
Prior myocardial infarction	18 (18)	444 (21.6)	0.393^a^
Left ventricular ejection fraction	*N* = 97	*N* = 1,992	0.746^b^
Normal (≥55%)	82 (82)	1,628 (79.1)	
Mildly reduced (45%–54%)	10 (10)	262 (12.7)	
Moderately reduced (30%–44%)	4 (4)	101 (4.9)	
Severely reduced (<30%)	1 (1)	1 (0)	

Data are shown as *n* (%) or mean ± standard deviation. Percentages for LVEF categories refer to the total cohort; *N* indicates available LVEF data. NSTE-ACS, non-ST-elevation acute coronary syndrome; PCI, percutaneous coronary intervention; STEMI, ST-elevation myocardial infarction.

a Chi-square test.

b Cochran-Armitage test for trend.

c Mann–Whitney *U* test.

d Percentage refers to diabetic/smoking patients only.

**Table 2:** Lesion analysis and characteristics of scaffold implantation: RMS vs. BIOSOLVE-IV.

Procedural characteristics		RMS (*N* = 104)	BIOSOLVE-IV (*N* = 2,141)	*p*-value
Lesion characteristics				
Lesion length (mm)		14.7 ± 5.0	14.8 ± 4.0	0.118^c^
Reference diameter (mm)		3.2 ± 0.7	3.2 ± 0.3	0.337^c^
Diameter stenosis (%)		87.7 ± 9.9	82.2 ± 10.6	<0.001^c^
Target vessel (%)	LAD	41 (39.4)	1,061 (49.6)	0.055^a^
	LCX	31 (29.8)	437 (20.4)	
	RCA	32 (30.8)	618 (28.9)	
	RI	0 (0)	25 (1.2)	
AHA/ACC (%)	Type A/B1	27 (26.0)	1,817 (84.9)	<0.001^a^
	Type B2/C	77 (74.0)	324 (15.1)	
Calcification	Moderate/severe	25 (24.0)	161 (7.5)	<0.001^a^
Tortuosity	Mild	99 (95.2)	1,878 (87.7)	0.068^a^
	Moderate	5 (4.8)	251 (11.7)	
	Severe	0 (0)	12 (0.6)	
Bifurcation		0 (0)	99 (4.6)	–
Thrombus		0 (0)	3 (0.1)	–
Chronic Total Occlusion		0 (0)	0 (0)	–
Characteristics of scaffold implantation				
Pre-dilation (%)		104 (100)	2,137 (99.8)	0.827^b^
Maximum applied pressure (atm)		15.5 ± 1.6	15.1 ± 3.4	0.064^c^
Scaffold length (mm)		18.3 ± 3.7	19.5 ± 3.9	0.002^c^
Scaffold diameter (mm)		3.1 ± 0.2	3.3 ± 0.2	<0.001^c^
Maximum implantation pressure (atm)		15.8 ± 2.1	14.4 ± 2.7	<0.001^c^
Post-dilation (%)		103 (99)	2,061 (95.7)	0.126^b^
Maximum applied pressure (atm)		16.4 ± 1.7	17.2 ± 3.2	<0.001^c^

Data are shown as mean ± standard deviation or *n* (%).

AHA/ACC, American Heart Association/American College of Cardiology; atm, atmosphere; LAD, left anterior descending artery; NC, non-compliant; RCA, right coronary artery; LCX, left circumflex artery; RI, ramus intermedius; ø, diameter.

a Chi-square test.

b Fisher's exact test.

c Mann–Whitney *U* test.

The incidence of TLF at 12 months, as well as its components TV-MI (target-vessel myocardial infarction) and CD-TLR (clinically-driven target-lesion revascularization), was numerically lower in RMS compared with BIOSOLVE-IV (5.0% vs. 5.4%; 2.0% vs. 2.3%; 4.0% vs. 4.5%, respectively). In contrast, the secondary endpoints cardiac death and scaffold thrombosis occurred less frequently in BIOSOLVE-IV (0.2% vs. 1.0%; 0.8% vs. 2.0%, respectively).

In the RMS registry, scaffold thrombosis occurred in two patients. One event occurred periprocedurally shortly after a technically challenging LAD intervention with atypical vessel origin, optimal scaffold sizing could not be fully achieved and thus, the 4P strategy was not satisfactorily fulfilled in this case. Repeat angiography revealed recurrent in-scaffold and distal thrombus formation with concomitant vasospasm, requiring repeated balloon angioplasty, adjunctive antithrombotic therapy with tirofiban, and DES implantation with scaffold overstenting. DAPT was escalated to ticagrelor because of suspected clopidogrel non-response, and the patient stabilized thereafter. The second event occurred on day 28 after scaffold implantation in the medial LCX and was classified as subacute scaffold thrombosis presenting as NSTEMI. During index PCI, the 4P strategy was fulfilled. IVUS at re-intervention demonstrated intraluminal scaffold struts extending towards the side-branch origin, resulting in a flow-limiting bifurcation-related thrombotic lesion. The lesion was successfully treated with DES implantation and bifurcation optimization, with satisfactory final angiographic and IVUS results. Antiplatelet therapy was escalated to aspirin and ticagrelor. Detailed case-level information is provided in Supplementary Table S5. Case-level review of the five TLF target lesions showed that four events occurred in AHA/ACC type B2 lesions and one event occurred in a type A lesion; no TLF event occurred in a type C lesion. The morphological features of these lesions were heterogeneous, without apparent clustering within a single complexity feature such as bifurcation, thrombus, ostial location, severe tortuosity, or chronic total occlusion. Given the small number of events, no formal outcome analysis according to individual lesion-complexity features was performed.

**Figure 3:**
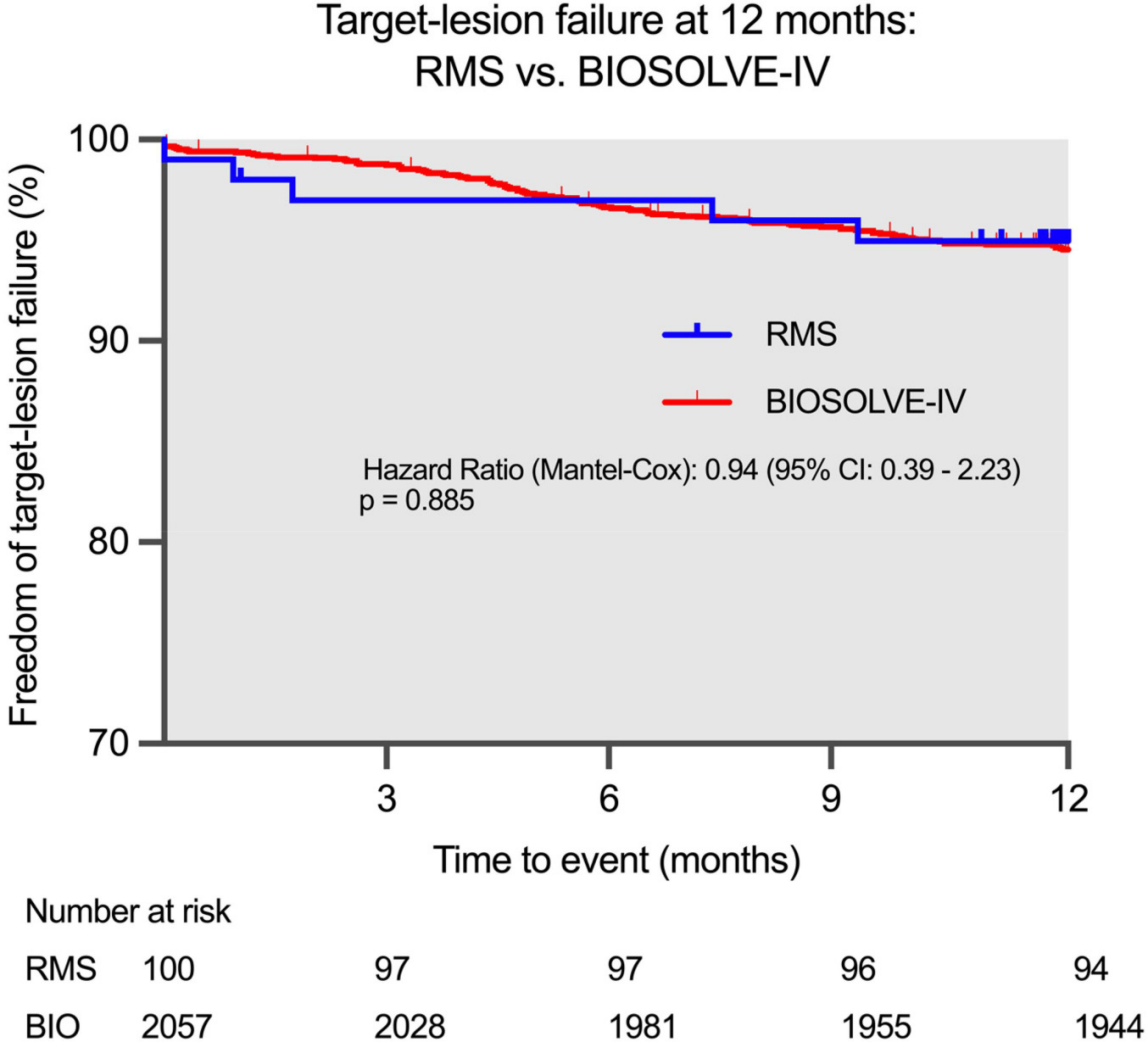
Kaplan–Meier curves for target-lesion failure (TLF) at 12 months in the RMS and BIOSOLVE-IV cohorts. Time-to-event analysis comparing freedom from target-lesion failure between RMS and BIOSOLVE-IV cohorts using the log-rank test. RMS, resorbable magnesium scaffold registry; BIOSOLVE-IV, BIOTRONIK post-marketing registry of the Magmaris scaffold; CI, confidence interval; HR, hazard ratio.

**Figure 4:**
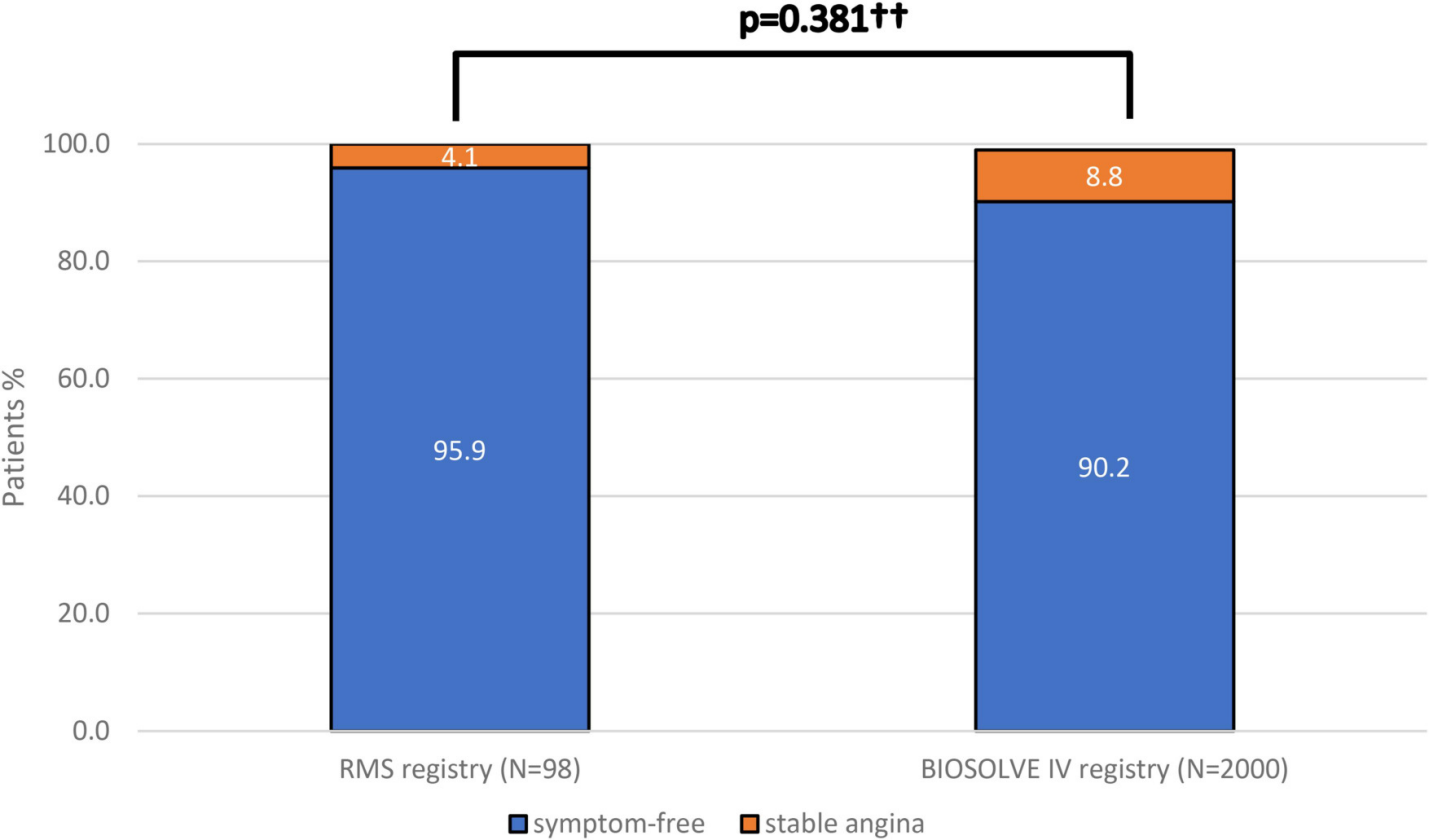
Anginal status at 12-month follow-up: RMS registry vs. BIOSOLVE-IV registry. Comparison of the proportion of symptom-free patients and those with stable angina at 12 months after Magmaris implantation. Unstable angina and silent ischemia are not shown due to very low incidence (≤0.5% each). ††: Exact test (Fisher–Freeman–Halton).

The unadjusted time-to-event analysis revealed no statistically significant differences between the two cohorts with regard to the primary endpoint TLF or any of the secondary endpoints. Patients in RMS had a 6% lower risk of TLF (Figure 3, Table 3). In RMS, slightly more than half of all patients were on DAPT at 12 months, whereas this was the case in more than two-thirds of patients in BIOSOLVE-IV (57.1% vs. 73.9%; *p* < 0.001). After 12 months following the index procedure, more than 90% of all patients in both cohorts were symptom-free. The distribution of ischemic status at 12 months did not differ significantly between the two cohorts (*p* = 0.381; Figure 4).

**Table 3:** Unadjusted outcome analysis: RMS vs. BIOSOLVE-IV.

Outcome analysis	RMS	BIOSOLVE-IV	*p*-value
Target-lesion failure	5 (5.0) [2.2–11.2]	112 (5.4) [4.5–6.5]	0.885
Cardiac death	1 (1.0) [0.2–5.5]	4 (0.2) [0.1–0.5]	0.099
Target-vessel MI	2 (2.0) [0.6–7.0]	47 (2.3) [1.7–3.0]	0.873
Clinically-driven TLR	4 (4.0) [1.6–9.8]	93 (4.5) [3.7–5.5]	0.851
Emergency CABG	0	0	–
All-cause mortality	2 (2.0) [0.6–7.0]	14 (0.7) [0.4–1.1]	0.130
Scaffold thrombosis (probable/definite)	2 (2.0) [0.6–7.0]	16 (0.8) [0.5–1.3]	0.384

Data are shown as *n* (Kaplan–Meier estimate %) [95% confidence interval]. *P*-value: log-rank test RMS vs. BIOSOLVE-IV. CABG, coronary artery bypass grafting; MI, myocardial infarction; TLR, target-lesion revascularization.

Baseline characteristics and outcomes in high-risk RMS subgroups, including patients with diabetes mellitus and NSTE-ACS, are summarized in Supplementary Tables S1–S4, showing no significant differences in target lesion failure or its individual components between these subgroups.

## 4 Discussion

This report represents the first scientific publication from the ongoing RMS registry, providing a direct statistical comparison with the BIOSOLVE-IV study. While RMS data were exclusively collected at the Cardiovascular Center Oberallgaeu-Kempten, Germany, BIOSOLVE-IV data were obtained from BIOTRONIK AG as part of a multicenter registry. Despite differences in baseline characteristics and lesion complexity between the two cohorts, the unadjusted analysis did not show statistically significant differences in the primary or secondary endpoints at 1 year. After 12 months following the index procedure, more than 90% of patients in both registries remained symptom-free. In BIOSOLVE-IV, the 2013 SCAI consensus definition was applied for periprocedural myocardial infarction, whereas the RMS registry used the ARC-2 criteria. The 2013 SCAI definition is known to be more sensitive but less specific for periprocedural MI compared with ARC-2, which may have artificially inflated the reported TLF rate in BIOSOLVE-IV relative to RMS. Applying the SCAI definition to the RMS population was not feasible because, in line with the real-world all-comers design of the registry, CK-MB measurements were available in only 24% of patients. Importantly, no relevant CK-MB elevations were observed in these cases. Moreover, given that postprocedural ECGs and clinical follow-up visits were obtained from all patients and none showed evidence of ischemia, the occurrence of undetected periprocedural myocardial infarction other than the STEMI appears highly unlikely.

Until today, bioresorbable scaffolds are not recommended for clinical use outside of clinical studies (6). Guideline recommendations, however, are almost exclusively derived from evidence obtained with polymer-based ABSORB scaffolds rather than magnesium-based devices. Nevertheless, adherence to current guidelines, meaning that bioresorbable scaffolds should still be implanted within the framework of clinical studies, remains essential until future randomized controlled trials (RCTs) may provide sufficient evidence to justify an update of the recommendations.

In our single-center analysis, the patient cohort was on average 3 years older than in BIOSOLVE-IV (*p* = 0.006). Well-established factors associated with an unfavorable prognosis regarding TLF, such as older age, a higher prevalence of NSTE-ACS, and a greater proportion of insulin-dependent diabetes mellitus, were more common in the RMS population. In BIOSOLVE-IV, multivariate analysis identified NSTE-ACS as the only independent predictor of an increased TLF risk (7). Thus, a higher prevalence of NSTE-ACS in BIOSOLVE-IV would have introduced a potential source of bias. Nevertheless, relevant differences in baseline characteristics and lesion morphology remained between the registries, and these differences must be considered when interpreting the between-group comparison.

Specific lesion characteristics were assessed according to the AHA/ACC classification of treated target lesions. Analysis demonstrated a significantly higher proportion of complex coronary lesions and of moderately to severely calcified lesions (Type B2/C) in RMS compared with BIOSOLVE-IV (*p* < 0.001). In BIOSOLVE-IV, a small number of bifurcation lesions (*n* = 99) were treated, whereas no bifurcation lesions were present in RMS. These cases, however, are likely captured within the B2/C category in BIOSOLVE-IV, as bifurcation involvement is a defining criterion of Type B lesions. Diameter stenosis was significantly greater in RMS than in BIOSOLVE-IV (87.7 ± 9.9% vs. 82.2 ± 10.6%; *p* < 0.001). In summary, target lesion treatment in RMS was procedurally more challenging than in BIOSOLVE-IV. Adherence to the 4P strategy was consistent in both registries. Nearly all lesions underwent pre-dilation before Magmaris implantation, while post-dilation compliance was higher in the RMS registry. Proper post-dilation of coronary scaffolds requires substantial operator experience, as excessive balloon pressure or overly aggressive expansion may result in serious complications such as scaffold deformation or dissection (19).

In BIOSOLVE-IV, many operators were first-time users of the Magmaris scaffold, which may have contributed to the omission of post-dilatation in some cases. Additional procedural differences included inflation pressures during scaffold implantation and post-dilatation. In RMS, Magmaris was implanted at significantly higher pressures, whereas post-dilatation pressures were higher in BIOSOLVE-IV. The mean post-dilatation pressure in RMS was 16.4 ± 1.7 atm and was therefore, on average, consistent with the currently recommended threshold of ≥16 atm. However, this value represents an average across lesions rather than a uniform procedural target in every individual case. In daily practice, operators aimed to achieve complete scaffold expansion and an acceptable final angiographic result while avoiding excessive mechanical stress on the scaffold, particularly in more complex lesions. Ultimately, it remains uncertain whether higher implantation pressure combined with lower post-dilatation pressure results in superior angiographic or clinical outcomes compared with the reverse strategy. It appears plausible that higher implantation pressure facilitates better initial scaffold apposition to the vessel wall, thereby reducing the necessity for high-pressure post-dilatation. This may also explain the observed differences in post-dilatation compliance between the two implantation strategies.

While DAPT after PCI with contemporary DES is well established and largely undisputed, this is not the case for BRS. To date, no randomized controlled trials have compared shorter vs. longer DAPT durations following BRS implantation, particularly for magnesium-based scaffolds. Given the substantially shorter resorption time and the presumed lower thrombogenicity of the magnesium alloy used in DREAMS compared with the PLLA backbone of ABSORB BVS, a DAPT duration of 6 months is now internationally considered adequate for magnesium scaffolds (18). At 12 months after the index procedure, 73.9% of patients in BIOSOLVE-IV remained on DAPT, compared with 56% of patients in the later RMS registry. Scaffold thrombosis was not the primary contributor to TLF in either registry; rather, CD-TLR accounted for most TLF events and was predominantly unrelated to thrombotic complications. Therefore, it can be assumed that DAPT duration did not have a relevant impact on TLF in this analysis. It is encouraging that more than 90% of patients in both cohorts were free from angina at 12-month follow-up. However, the open-label design of both studies and the potential influence of concomitant antianginal therapy must be considered when interpreting these results.

Time-to-event analyses for both primary and secondary endpoints revealed no statistically significant differences. The univariate, unadjusted outcome analysis showed no statistically significant difference in TLF risk. Cardiac deaths, scaffold thrombosis, and overall mortality occurred numerically more often in RMS, whereas target-vessel myocardial infarction and clinically driven target-lesion revascularization were more frequent in BIOSOLVE-IV; none of these differences reached statistical significance. These observations may partly reflect the greater clinical and angiographic complexity of the RMS cohort. Given the relatively small sample size, single events can substantially affect reported percentages, particularly the two scaffold thrombosis cases, which represent 2% of the entire cohort. As a single-center analysis, the RMS registry is also inherently more susceptible to random variation. Nevertheless, despite higher age and greater lesion complexity in RMS, TLF and CD-TLR did not differ significantly between the two cohorts. Propensity-score adjustment or multivariable modeling was considered but deemed inappropriate given the limited number of events and the exploratory intent of the analysis.

In the Italian Magmaris Registry (207 patients with 209 lesions), 23% of patients presented with an acute coronary syndrome at enrollment. Forty-six percent of treated target lesions were classified as Type B2/C, and a bifurcation lesion was present in approximately one-third of cases. The investigators demonstrated high adherence to the 4P strategy. Notably, in cases with uncertainty regarding lesion characteristics based on visual or quantitative coronary analysis (QCA), intravascular imaging using IVUS or OCT was employed to ensure optimal vessel sizing. Only one case of scaffold thrombosis occurred within 12 months. The TLF rate was 5.4%, comparable to that observed in RMS and BIOSOLVE-IV (9).

Intravascular imaging now holds a Class I (Level of evidence A) recommendation in the guidelines for revascularization of complex coronary lesions in chronic coronary syndrome (20). However, it was not mandatory in RMS and BIOSOLVE-IV and its role in BRS implantation strategies remains controversial. The investigators of the HONEST trial compared OCT vs. angiography guided Magmaris implantation in a NSTE-ACS cohort and found no benefit in coronary healing but an increased rate of post-dilatation and acquired scaffold malapposition in the OCT group (21). Repetitive post-dilatation may increase mechanical stress on magnesium scaffolds and potentially predispose to device deformation or fracture. The lesson learned from this trial is that intravascular imaging may be important for vessel sizing, but it must be used with caution and rigorously optimizing the final angiographic result based on OCT imaging should be avoided. Given the lesion complexity, the RMS cohort would retrospectively have been ideal for intravascular imaging. Whether its systematic use would have resulted in differences in clinical outcomes remains for speculation.

Outcomes of published Magmaris trials are encouraging, although contemporary DES remain the standard of care and the most relevant clinical benchmark for complex coronary lesions. In a large individual patient-data pooled analysis of seven randomized trials including patients treated with second-generation DES, ACC/AHA type B2/C lesions were associated with higher 1-year TLF rates than type A/B1 lesions (4.6% vs. 3.0%), confirming the continued prognostic relevance of lesion complexity even in the contemporary DES era (22). In this context, the 5.0% TLF rate observed in the RMS cohort appears clinically relevant, particularly given that 74.0% of treated lesions were classified as type B2/C. While these data are not directly comparable because of differences in design, population, and device platform, they provide a useful contemporary reference for interpreting the present real-world scaffold findings.

In April 2020, the BIOMAG-I trial was initiated as a first-in-human study investigating the successor of Magmaris, the Freesolve scaffold (DREAMS 3G). Compared with Magmaris, this third-generation device features thinner struts, greater radial strength, an expanded range of available sizes, and enhanced radiopacity. Notably, these technical refinements resulted in a 52% reduction in late lumen loss at 6 months and a 38% reduction at 12 months compared with Magmaris, achieving non-inferiority to contemporary DES. It should be emphasized, however, that these findings are based on only 116 patients with 117 lesions. Clinical outcomes after 12 months were favorable, with a TLF rate of 2.9% and no reported cases of scaffold thrombosis or target-vessel myocardial infarction (18). Recently published 3-year results from BIOMAG-I indicate sustained clinical stability beyond the scaffold resorption period, with only one additional TLF event and no reported cardiac death, target-vessel myocardial infarction, or definite/probable scaffold thrombosis up to 3 years (23).

The RCT BIOMAG-II (Freesolve vs. Xience everolimus-eluting stent) is currently ongoing (24). Its results will be pivotal for the future of bioresorbable scaffold technology and may determine whether a paradigm shift in the interventional management of *de novo* coronary lesions is forthcoming. The *leave nothing behind* concept could thus gain renewed clinical relevance, potentially expanding the therapeutic armamentarium in coronary revascularization.

### 4.1 Limitations

Our analysis has several important clinical and statistical limitations.

#### 4.1.1 Limitations of cross-registry comparison from a clinical point of view

Both registries were conducted at different times. Negative experiences from the BIOSOLVE-IV registry may have already been considered by investigators from RMS. Event adjudication in RMS was performed by a cardiology resident under supervision of experienced interventional cardiologists. Although not conducted by an independent clinical events committee, this approach minimized potential observer bias. Moreover, the RMS cohort consisted exclusively of patients treated at Cardiovascular Center Oberallgaeu-Kempten. Consequently, the present analysis may reflect local patient selection, implantation technique, and follow-up practice, which limits external validity in comparison with a multicentre registry. The low use of intravascular imaging is a limitation in this complex-lesion cohort. IVUS/OCT was not mandatory in the RMS registry and was performed at the operator's discretion. Because no systematic angiographic or intravascular imaging follow-up was conducted, mechanistic conclusions regarding scaffold expansion, malapposition, restenosis, or resorption cannot be drawn. Even in BIOMAG II and BIOMAG III, intravascular imaging is still not mandatory because of feasibility and practicability in daily clinical use and because it may also have adverse effects like overtreatment of plaques beyond the culprit lesions that do not cause ischemia or treatment of local dissections that are not flow limiting. Anyway, we do emphasize that intravascular imaging should be carried out more generously in selected critical cases. With IVUS/OCT used in only 2 patients, an outcome comparison by imaging use was not feasible.

#### 4.1.2 Limitations of cross-registry comparison from a statistical point of view

The main drawback is a smaller sample size of RMS (*n* = 100) compared to BIOSOLVE-IV (*n* = 2057). No formal sample size calculation was performed, as this was an exploratory, hypothesis-generating analysis. Consequently, the study was not powered to draw formal safety or efficacy conclusions, and confidence intervals were wide. Comparison of TLF rates between RMS and BIOSOLVE-IV using log-rank test must be interpreted with caution due to differences in baseline characteristics. While certain variables differed statistically, only differences in ischemic status as well as lesion calcification and complexity are likely to be clinically relevant. Multivariable Cox regression or propensity-score matching would have been appropriate, but was not feasible due to limited number of events and smaller cohort size in RMS. Therefore, the absence of statistically significant differences between registries should not be interpreted as evidence of equivalence, but rather as a finding from an exploratory, unadjusted comparison. Nevertheless, the present analysis provides descriptive real-world data on the use of bioresorbable magnesium scaffolds in patients with predominantly complex coronary lesions.

## 5 Conclusion

Our data suggest that acceptable 1-year outcomes with Magmaris can be achieved even in a real-world population with predominantly complex coronary lesions. Although Magmaris has since been succeeded by Freesolve, the present analysis remains clinically relevant by providing a reference point for the interpretation and evaluation of newer magnesium scaffold technologies.

## Data Availability

The original contributions presented in the study are included in the article/Supplementary Material, further inquiries can be directed to the corresponding author.
