# Supplementary material for: One-year outcomes of bioresorbable magnesium scaffold implantation in complex coronary lesions
Source: Front Cardiovasc Med. 2026 Jun 12;13:1854686. doi: 10.3389/fcvm.2026.1854686 (PMC13306953; doi:10.3389/fcvm.2026.1854686)
Supplement: Supplementary file 1 [file Table1.docx]

### Supplementary data

**One-year outcomes of bioresorbable magnesium scaffold implantation in complex coronary lesions**

Simon Wölbert¹˒³ · Thomas Schmidt¹ · Fabian Wittek¹ · Benjamin Mayer² · Philip Raake³ · Dario Bongiovanni^3^ · Jan Torzewski¹

¹ Cardiovascular Center Oberallgäu-Kempten, Kempten, Germany

² Institute of Epidemiology and Medical Biometry, University of Ulm, Ulm, Germany

³ Department of Internal Medicine I, University Hospital Augsburg, Augsburg, Germany

Corresponding author:

Jan Torzewski

Cardiovascular Center Oberallgaeu-Kempten, Germany

Email: [jan.torzewski@klinikverbund-allgaeu.de](mailto:jan.torzewski@klinikverbund-allgaeu.de)

**Supplementary Table 1:** Baseline characteristics of subgroups in RMS (patient level). Comparison of demographic and clinical parameters according to the presence of NSTE-ACS, diabetes mellitus, and lesion type (B2/C vs. A/B1) in the RMS registry.

|  | **NSTE-ACS** | | | **Diabetes mellitus** | | | **Type B2/C lesions** | | |
| --- | --- | --- | --- | --- | --- | --- | --- | --- | --- |
|  | Yes  N=26 | No  N=74 | p-value | Yes  N=26 | No  N=74 | p-value | Yes  N=73 | No  N=27 | p-value |
| Age | 62.3 ± 7.3 | 65.8 ± 8.7 | 0.071* | 65.5 ± 8.7 | 64.8 ± 8.2 | 0.888** | 65.2 ± 8.2 | 64.1 ± 9.3 | 0.583* |
| Male | 20 (77.0) | 58 (78.4) | 0.879° | 23 (88.5) | 55 (74.3) | 0.134° | 58 (79.5) | 20  (74.1) | 0.564° |
| Arterial Hypertension | 19 (73.1) | 54 (73.0) | 0.992° | 21 (80.8) | 52 (70.3) | 0.300° | 54 (74) | 19 (70.4) | 0.719° |
| Dyslipidaemia | 12 (46.2) | 37 (50.0) | 0.736° | 15 (57.7) | 34 (45.9) | 0.303° | 35 (47.9) | 14 (51.9) | 0.729° |
| Diabetes mellitus | 6 (23.1) | 20 (27.0) | 0.693° | 26 (100) | 0 (0) | - | 20 (27.4) | 6 (22.2) | 0.600° |
| Chronic kidney disease | 1 (3.8) | 3 (4.1) | 1.000°° | 1 (3.8) | 3 (4.1) | 1.000°° | 3 (4.1) | 1 (3.7) | 1.000°° |
| Smoking history | 14 (53.8) | 44 (59.5) | 0.899° | 21 (80.8) | 37 (50) | 0.006° | 42 (57.5) | 16 (59.3) | 0.877° |
| Obesity | 11 (42.3) | 29 (39.2) | 0.780° | 16 (61.5) | 24 (32.4) | 0.009° | 30 (41.1) | 10 (37) | 0.713° |
| Positive family history | 9 (34.6) | 31 (41.9) | 0.515° | 9 (34.6) | 31 (41.9) | 0.515° | 31 (42.5) | 9 (33.3) | 0.408° |
| Coronary artery disease | 5 (19.2) | 31 (41.9) | 0.038°° | 12 (46.2) | 24 (32.4) | 0.210° | 24 (32.9) | 12 (44.4) | 0.285° |
| Myocardial infarction | 1 (3.8) | 17 (23.0) | 0.036° | 4 (15.4) | 14 (18.9) | 0.775°° | 13 (17.8) | 5 (18.5) | 1.000°° |
| PTCA/Stent | 6 (23.1) | 29 (39.2) | 0.138° | 12 (46.2) | 19 (25.7) | 0.166° | 23 (31.5) | 12 (44.4) | 0.229° |
| COPD | 2 (7.7) | 5 (6.8) | 1.000°° | 3 (11.5) | 4 (5.4) | 0.372°° | 6 (8.2) | 1 (3.7) | 0.671°° |
| Heart failure | 5 (19.2) | 14 (18.9) | 1.000°° | 7 (26.9) | 12 (16.2) | 0.253°° | 14 (19.2) | 5 (18.5) | 0.409° |

Data are shown as mean ± standard deviation, [95% confidence interval] or n (%); **Abbreviations: COPD = Chronic Obstructive Pulmonary Disease; LAD = Left Anterior Descending Artery; MLD = Minimal Lumen Diameter;** NSTE-ACS = non-ST-elevation acute coronary syndrome**; PTCA = Percutaneous Transluminal Coronary Angioplasty; RCA = Right Coronary Artery; RCX = Ramus Circumflexus; RI = Ramus Intermedius. Statistical tests: * = Chi² test; ° = Fisher’s exact test; ** = Student’s t-test; °° = Mann–Whitney U-test**

**Supplementary Table 2:** Lesion characteristics of subgroups in RMS (lesion level). Comparison of angiographic lesion parameters according to the presence of NSTE-ACS, diabetes mellitus, and lesion type (B2/C vs. A/B1) in the RMS registry.

|  | **NSTE-ACS** | | | **Diabetes mellitus** | | | **Type B2/C lesions** | | |
| --- | --- | --- | --- | --- | --- | --- | --- | --- | --- |
| **All lesions** | Yes  N=27 | No  N=77 | p-value | Yes  N=27 | No  N=77 | p-value | Yes  N=77 | No  N=27 | p-value |
| Lesion length  (mm) | 13.9 ± 4.2 | 14.9 ± 5.2 | 0.326** | 13.8 ± 4.6 | 14.8 ± 4.8 | 0.296** | 16.0 ± 5.0 | 11.0 ± 2.6 | <0.001** |
| Reference  diameter (mm) | 3.3 ± 0.8 | 3.2 ± 0.7 | 0.813** | 3.5 ± 0.8 | 3.2 ± 0.7 | 0.081** | 3.2 ± 0.7 | 3.2 ± 0.7 | 0.711** |
| Diameter stenosis (%) | 91.3 ± 5.9 | 86.4 ± 10.7 | 0.045** | 86.8 ± 8.6 | 88.2 ± 10.3 | 0.202** | 88.1 ± 9.3 | 86.5 ± 11.4 | 0.932** |
| **Target vessel (%)**  LAD  RCX  RCA | 7 (25.9)  12 (44.4)  8 (29.6) | 34 (44.2)  19 (24.7)  24 (31.2) | 0.115 ° | 8 (29.6)  8 (29.6)  11 (40.7) | 33 (42.9)  23 (29.9)  21 (27.3) | 0.356° | 32 (41.6)  18 (23.4)  27 (35.1) | 9 (33.3)  13 (48.1)  5 (18.5) | 0.044° |
| **AHA/ACC**  Type A  Type B1  Type B2  Type C | 0  11 (40.7)  12 (44.4)  4 (14.8) | 5 (6.5)  11 (14.3)  51 (66.2)  10 (13.0) | 0.019° | 3 (11.1)  3 (11.1)  18 (66.7)  3 (11.1) | 2 (2.6)  19 (24.7)  45 (58.4)  11 (14.3) | 0.185° | 0  0  63 (81.8)  14 (18.2) | 5 (18.5)  22 (81.5)  0  0 | - |
| **Calcification**  none  mild  moderate  severe | 5 (18.5)  17 (63.0)  5 (18.5)  0 | 8 (10.4)  49 (63.6)  17 (22.1)  3 (3.9) | 0.526° | 2 (7.4)  19 (70.4)  6 (22.2)  0 | 11 (14.3)  47 (61)  16 (20.8)  3 (3.9) | 0.551° | 3 (3.9)  49 (63.6)  22 (28.6)  3 (3.9) | 10 (37.0)  17 (63.0)  0  0 | <0.001° |
| **Tortuosity**  mild  moderate  severe | 25 (92.6)  2 (7.4)  0 | 74 (96.1)  3 (3.9)  0 | 0.603° | 26 (96.3)  1 (3.7)  0 | 73 (94.8)  4 (5.2)  0 | 1.000° | 72 (93.5)  5 (6.5)  0 | 27 (100)  0  0 | 0.323° |
| **Symmetry**  concentric  eccentric | 8 (29.6)  19 (70.4) | 15 (19.5)  62 (80.5) | 0.290° | 6 (22.2)  21 (77.8) | 17 (22.1)  60 (77.9) | 1.000° | 2 (2.6)  75 (97.4) | 21 (77.8)  6 (22.2) | <0.001° |
| **Vessel contour**  smooth  irregular | 19 (70.4)  8 (29.6) | 30 (39.0)  47 (61.0) | 0.007° | 13 (48.1)  14 (51.9) | 36 (46.8)  41 (53.2) | 0.901° | 22 (28.6)  55 (71.4) | 27 (100)  0 | <0.001° |
| MLD before predilatation (mm) | 0.27 ± 0.17 | 0.45 ± 0.36 | 0.042** | 0.46 ± 0.32 | 0.38 ± 0.33 | 0.095** | 0.39 ± 0.31 | 0.45 ± 0.41 | 0.753** |
| MLD after predilatation (mm) | 2.40 ± 0.80 | 2.38 ± 0.57 | 0.689** | 2.56 ± 0.66 | 2.34 ± 0.63 | 0.128** | 2.40 ± 0.64 | 2.38 ± 0.64 | 0.697** |
| Acute gain | 2.97 ± 0.77 | 2.74 ± 0.64 | 0.281** | 2.97 ± 0.71 | 2.76 ± 0.67 | 0.176** | 2.01 ± 0.69 | 1.92 ± 0.64 | 0.979** |
| Diameter stenosis after predilatation | 25.9 ± 12.5 | 25.4 ± 11 | 0.604** | 25.4 ± 11.6 | 25.8 ± 11.4 | 0.987** | 25 ± 11.8 | 27 ± 10.7 | 0.302** |

Data are shown as mean ± standard deviation, [95% confidence interval] or n (%); **Abbreviations:** AHA/ACC = American Heart Association/American College of Cardiology; LAD = Left Anterior Descending Artery; MLD = Minimal Lumen Diameter; NSTE-ACS = non-ST-elevation acute coronary syndrome; RCA = Right Coronary Artery; RCX = Ramus Circumflexus; RI = Ramus Intermedius. **Statistical tests:** * = Chi² test; ° = Fisher’s exact test; ** = Student’s t-test; **°°** = Mann–Whitney U-test

**Supplementary Table 3:** Implantation characteristics of subgroups in RMS (lesion level).
Comparison of implantation parameters according to the presence of NSTE-ACS, diabetes mellitus, and lesion type (B2/C vs. A/B1) in the RMS registry.

|  | **NSTE-ACS** | | | **Diabetes mellitus** | | | **Typ B2/C lesions** | | |
| --- | --- | --- | --- | --- | --- | --- | --- | --- | --- |
|  | Yes  N=27 | No  N=77 | p-value | Yes  N=27 | No  N=77 | p-value | Yes  N=77 | No  N=27 | p-value |
| Predilatation performed (%) | 27 (100) | 77  (100) | - | 27  (100) | 77 (100) | - | 77  (100) | 27 (100) | - |
| Maximum applied pressure (atm) | 14.1 ± 2.4 | 14.8 ± 1.9 | 0.225** | 15.4 ± 1.4 | 14.8 ± 1.9 | 0.246** | 15.1 ±  1.8 | 14.5 ± 1.9 | 0.083** |
| Duration of predilatation (s) | 9.6 ± 4.8 | 9.3 ±  6.1 | 0.621** | 7.9 ± 4.3 | 9.9 ± 6.1 | 0.083** | 8.5 ±  5.5 | 12.0 ± 5.6 | 0.002** |
| NC-Balloon Ø (mm) | 3.1 ± 0.3 | 3.1 ±  0.3 | 0.364** | 3.1 ± 0.3 | 3.1 ± 0.3 | 0.736** | 3.1 ±  0.3 | 3.0 ± 0.3 | 0.751** |
| Scaffold length (mm) | 18.9 ±  4.2 | 18.1 ±  3.5 | 0.399** | 18.5 ± 3.6 | 18.1 ± 3.7 | 0.565** | 18.6 ±  3.7 | 17.0 ± 3.2 | 0.046** |
| Scaffold diameter (mm) | 3.2 ±  0.3 | 3.1 ±  0.2 | 0.058** | 3.2 ± 0.2 | 3.1 ± 0.2 | 0.654** | 3.1 ±  0.2 | 3.2 ± 0.2 | 0.654** |
| Maximum applied pressure (atm) | 15.6 ±  2.1 | 15.9 ±  2.2 | 0.530** | 16.5 ± 1.1 | 15.5 ± 2.4 | 0.078** | 16.0 ± 1.8 | 15.0 ± 3.0 | 0.137** |
| Duration of implantation (s) | 22.0 ±  3.1 | 22.4 ±  5.0 | 0.493** | 21.5 ± 3.1 | 22.4 ± 4.9 | 0.377** | 22.1 ±  4.7 | 22.3 ± 3.9 | 0.890** |
| Postdilatation performed (%) | 27 (100) | 66  (98.5) | 1.000°° | 27  (100) | 76 (98.7) | 1.000°° | 76  (98.7) | 27 (100) | 1.000°° |
| Maximum applied pressure (atm) | 16.6 ± 1.4 | 16.6 ± 1.4 | 0.777** | 16.9 ±  1.2 | 16.5 ± 1.5 | 0.263** | 16.6 ±  1.4 | 16.6 ± 1.4 | 0.516** |
| Duration of postdilatation (s) | 9.9 ± 7.1 | 8.2 ±  7.2 | 0.024** | 6.3 ± 4.1 | 9.5 ± 7.6 | 0.007** | 7.6 ±  4.4 | 11.6 ± 7.7 | 0.028** |
| NC-Balloon Ø | 3.2 ± 0.3 | 3.1 ±  0.3 | 0.194** | 3.2 ± 0.3 | 3.2 ±  0.3 | 0.785** | 3.1 ±  0.3 | 3.2 ± 0.3 | 0.104** |

Data are shown as mean ± standard deviation, [95% confidence interval] or n (%); **Abbreviations:** NC = non-compliant; NSTE-ACS = non-ST-elevation acute coronary syndrome; atm = atmosphere; Ø = diameter. **Statistical tests:** * = Chi² test; ° = Fisher’s exact test; ** = Student’s t-test; °° = Mann–Whitney U-test.

**Supplementary Table 4:** Outcome analysis in high-risk subgroups (RMS)

| **Outcome** | **Full cohort** | **Diabetes mellitus** | **p-value** | **NSTE-ACS** | **p-value** |
| --- | --- | --- | --- | --- | --- |
| Target-lesion failure | 5 (5.0) [2.2–11.2] | 1 (3.8) [0.7–18.9] | 0.779 | 0 (0) [0.0–9.9] | 0.094 |
| Cardiac death | 1 (1.0) [0.2–5.5] | 0 (0) [0.0–12.9] | 0.561 | 0 (0) [0.0–9.9] | 0.460 |
| Target-vessel MI | 2 (2.0) [0.6–7.0] | 1 (3.8) [0.7–18.9] | 0.442 | 0 (0) [0.0–9.9] | 0.298 |
| Clinically-driven TLR | 4 (4.0) [1.6–9.8] | 1 (3.8) [0.7–18.9] | 0.977 | 0 (0) [0.0–9.9] | 0.134 |
| All-cause death | 2 (2.0) [0.6–7.0] | 0 (0) [0.0–12.9] | 0.430 | 0 (0) [0.0–9.9] | 0.298 |
| Clinically-driven TVR | 4 (4.0) [1.6–9.8] | 1 (3.8) [0.7–18.9] | 0.977 | 0 (0) [0.0–9.9] | 0.298 |
| Scaffold thrombosis (definite/probable) | 2 (2.0) [0.6–7.0] | 1 (3.8) [0.7–18.9] | 0.442 | 0 (0) [0.0–9.9] | 0.298 |

**Abbreviations:** MI = myocardial infarction; NSTE-ACS = non-ST-elevation acute coronary syndrome; TLR = target-lesion revascularization; TVR = target-vessel revascularization. **Statistical note:** Data are shown as n (Kaplan–Meier estimator) [95% confidence interval]. p-values refer to log-rank tests comparing diabetic vs. non-diabetic patients and NSTE-ACS vs. non-NSTE-ACS subgroups.

**Supplementary Table 5:** Target lesion failure event characteristics in RMS

| **Case** | **Event** | **Time since intervention (d)** | **Age (years)** | **Diabetes** | **Ischemic status*** | **Target vessel** | **AHA/ACC** | **Calcification** | **4P** | **DAPT**** | **Event description** |
| --- | --- | --- | --- | --- | --- | --- | --- | --- | --- | --- | --- |
| 1 | SCT-TVMI | 0 | 71 | X | SAP | LAD | B2 | m | X | √ | STE due to periprocedural scaffold thrombosis, suspected clopidogrel failure, switch from clopidogrel to ticagrelor, stabilization and discharge on day 5 post-intervention, solved with 2×DES (Synergy 3.0×22 mm, Synergy 2.0×20 mm). |
| 2 | SCT-TVMI | 28 | 64 | √ | SAP | RCX | A | m | √ | √ | Hospitalization with NSTE-ACS, IVUS-guided revascularization with proof of intraluminal scaffold struts and thrombus, solved with 2×DES (Synergy 3.0×26 mm + 3.0×22 mm). |
| 3∫ | NCD | 31 | 77 | √ | SI | LAD | B2 | s | X | √ | Hospitalization with haemorrhagic shock due to upper gastrointestinal bleeding (6 PRBCs transfused), aspiration pneumonia, chronic renal failure; non-cardiac death due to multiorgan failure; no evidence for causal relation to Magmaris. |
| 4 | CD | 52 | 63 | X | SAP | LAD | B2 | m | X | √ | Unwitnessed death, no autopsy performed; causal relation to Magmaris could not be excluded, therefore counted as TLF due to cardiac death. |
| 5 | CD-TLR | 222 | 78 | X | UAP | RCA | B2 | m | √ | √ | Hospitalization via GP due to unstable angina, angiography revealed in-scaffold stenosis (99 % medial RCA), additional ostial RCA stenosis (80 %), solved with 1×DES medial RCA (Promus 3.5×28 mm) and 1×DES ostial RCA (Synergy Megatron 4.0×16 mm). |
| 6 | CD-TLR | 281 | 68 | X | SI | RCA | B2 | m | X | X | No clinical symptoms, routine follow-up (stress test) revealed ST-depression; elective angiography showed in-scaffold stenosis (80 %), solved with 1×DES (Synergy 3.0×22 mm). |

**Abbreviations:** CD = cardiac death; CD-TLR = clinically-driven target lesion revascularization; d = days; DES = drug-eluting stent; GP = general practitioner; IVUS = intravascular ultrasound; LAD = left anterior descending artery; m = none/mild; NCD = non-cardiac death; NSTE-ACS = non-ST-elevation acute coronary syndrome; RCA = right coronary artery; RCX = ramus circumflexus; s = moderate/severe; sAP = stable angina; ScT = scaffold thrombosis; SI = silent ischemia; STEMI = ST-elevation myocardial infarction; TVMI = target-vessel myocardial infarction; uAP = unstable angina; ∫ = no target lesion failure; * = at baseline; ** = at the time of event

**Supplementary Table 6:** Stepwise analysis of coronary lesion morphology using FUJIFILM Synapse PACS Viewer (version 5.7.242)

| Type A lesion | Type C lesion |
| --- | --- |
| 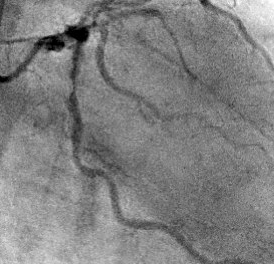  1a  RAO, caudal* | 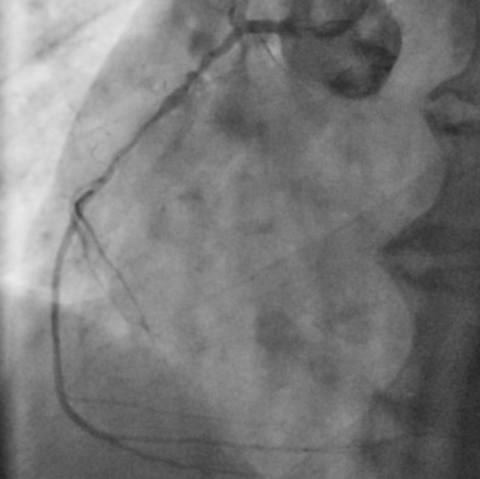  2a  LAO, cranial* |
| 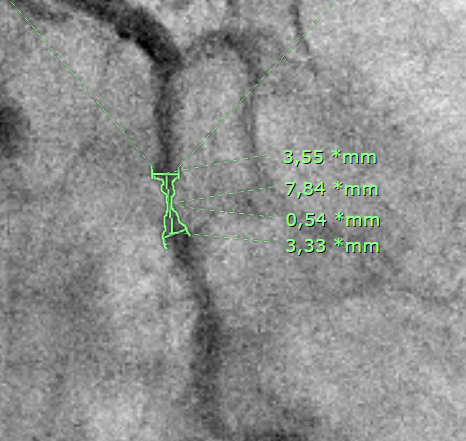  1b  RAO, caudal* | 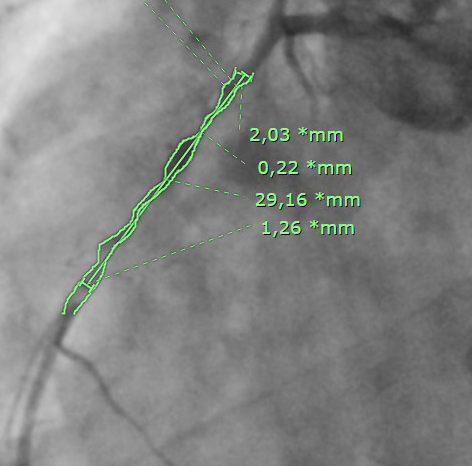  2b  LAO, cranial* |
| 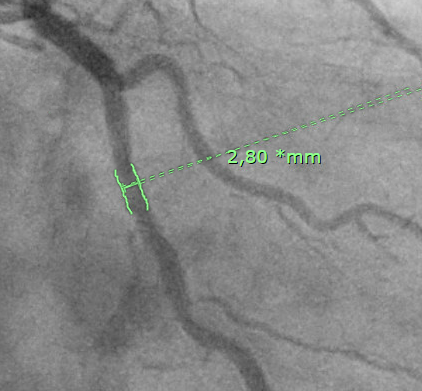  RAO, caudal*  1c | 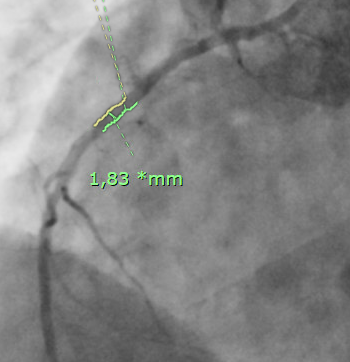  2c  LAO, cranial* |
| 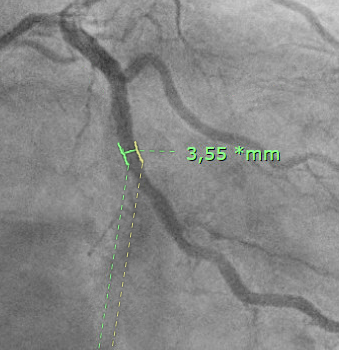  RAO, caudal*  1d | 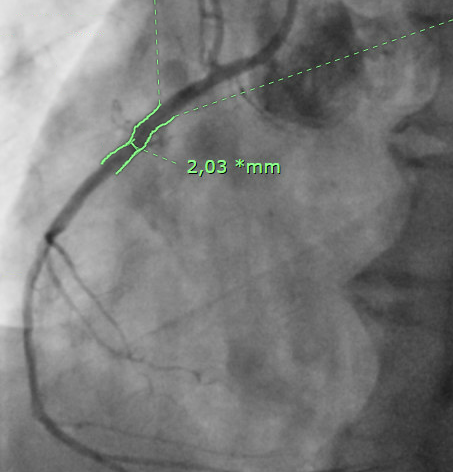  LAO, cranial*  2d |
| 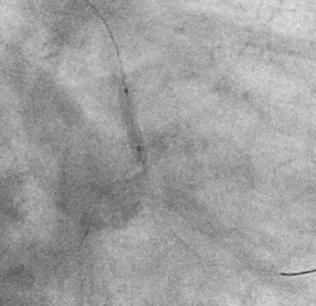  RAO, caudal*  1e | 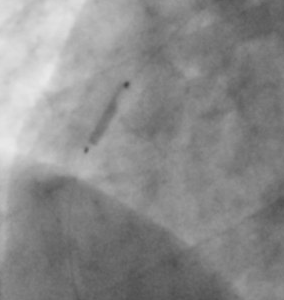  LAO, cranial*  2e |
| **Figure 1a:** Lesion in medial circumflex artery (RCX) **Figure 1b:** Measurement of lesion length, RD, MLD **Figure 1c:** Measurement of MLD after predilatation **Figure 1d:** Measurement of MLD after postdilatation **Figure 1e:** Complete balloon expansion during predilatation. | **Figure 2a:** Lesion in proximal-medial RCA **Figure 2b:** Measurement of lesion length, RD, MLD **Figure 2c:** Measurement of MLD after predilatation **Figure 2d:** Measurement of MLD after postdilatation **Figure 2e:** Focally incomplete balloon expansion during predilatation (calcification) |

**Abbreviations:** RD = reference diameter; RCX = Ramus Circumflexus; MLD = minimal lumen diameter; RCA = Right Coronary Artery; * = projection; RAO = right anterior oblique; LAO = left anterior oblique.

**Supplementary File 1: Cochran-Armitage test for trends – Rstudio script**

# --- Creation of matrix with frequencies of LVEF ---

lvef_data <- matrix(c(

82, 1628, # normal LVEF (RMS, BIOSOLVE)

10, 262, # mildly reduced

4, 101, # moderately reduced

1, 1 # severely reduced

), nrow = 4, byrow = TRUE)

# --- Create rows and columns ---

rownames(lvef_data) <- c("Normal", "Mildly reduced", "Moderately reduced", "Severely reduced")

colnames(lvef_data) <- c("RMS", "BIOSOLVE")

# --- Visualize table ---

print(lvef_data)

# --- Cochran-Armitage test ---

CochranArmitageTest(lvef_data)

# Notes:

# ¹ normal ejection fraction (≥55%)

# ² mildly reduced ejection fraction (45–54%)

# ³ moderately reduced ejection fraction (30–44%)

# ⁴ severely reduced ejection fraction (<30%)

# Tested with R version 4.4.1 and DescTools 0.99.56
